# High Quality Monolayer Graphene Synthesized by Resistive Heating Cold Wall Chemical Vapor Deposition

**DOI:** 10.1002/adma.201501600

**Published:** 2015-06-05

**Authors:** Thomas H. Bointon, Matthew D. Barnes, Saverio Russo, Monica F. Craciun

**Affiliations:** ^1^ Centre for Graphene ScienceCollege of EngineeringMathematics and Physical SciencesUniversity of ExeterExeterEX4 4QLUK

**Keywords:** chemical vapor deposition, graphene, resistive heating, touch sensors

## Abstract

**The growth of graphene** using resistive‐heating cold‐wall chemical vapor deposition (CVD) is demonstrated. This technique is 100 times faster and 99% lower cost than standard CVD. A study of Raman spectroscopy, atomic force microscopy, scanning electron micro­scopy, and electrical magneto‐transport measurements shows that cold‐wall CVD graphene is of comparable quality to natural graphene. Finally, the first transparent flexible graphene capacitive touch‐sensor is demonstrated.

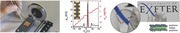

Chemical vapor deposition (CVD) of monolayer graphene on copper^1^, ^2^ has emerged as one of the most competitive growth methods for securing the industrial exploitation of graphene, due to its compatibility with Si and roll‐to‐roll technologies.^3^ Recently, there has been tremendous progress in controlling the morphology,^4^, ^5^, ^6^ functionalization,^7^, ^8^, ^9^, ^10^ and growth of heterostructures of intrinsic and doped graphene.^11^ However, the low‐throughput and the very high production cost for high‐quality CVD graphene are central challenges for the industrial exploitation of this material.^12^, ^13^ The most common CVD approach is to use a hot‐wall system where Cu foils are heated at temperatures ≈1000 °C in a quartz tube furnace through which the precursor hydrocarbon gas flows. The long processing time, that can take a few hours, limits the throughput of graphene by this method. At the same time the typical cost of graphene produced in this way is in excess of 1 cm^−2^, whereas its retail price ranges from 4.57 cm^−2^ to 21 cm^−2^ (see the Supporting Information). Therefore, a way forward to increase the throughput and reduce the production cost is to grow graphene in a cold wall CVD system which heats selectively only the Cu foils. Few types of cold wall CVD have been investigated so far for the growth of graphene^3^, ^14^, ^15^, ^16^, ^17^, ^18^, ^19^ such as magnetic induction heating CVD,^14^ rapid thermal annealing CVD using halogen lamp heating,^15^, ^16^ Joule heating CVD,^17^, ^18^ and resistively heated stage CVD.^19^ Of all these methods, the resistively heated stage CVD approach allows for faster, more efficient heating and cooling, shorter growth time, and less gas consumption. This method provides a more uniform substrate heating, it reduces the chemical reactions which can take place in the gas phase at high temperature known to contaminate graphene and it allows for very fast cooling rates, which have been shown to enhance the quality of graphene grown by CVD on copper foil.^20^ Furthermore, this type of cold‐wall CVD system is found in manufacturing plants of the semiconductor industries. Most importantly we show that with this method truly high quality monolayer graphene can be reproducibly grown. To date, virtually nothing is known on the growth mechanism of monolayer graphene by cold‐wall CVD, as well as on its quality and suitability for flexible electronic applications. Therefore, understanding the growth and properties of graphene obtained with cold‐wall CVD is imperative to enable the exploitation of this material and facilitate the birth of novel graphene‐based applications.

Here we report a completely new mechanism for the growth of graphene by resistively heated stage cold‐wall CVD which is markedly different from the growth mechanism of graphene in a hot‐wall CVD. Through a combined study of Raman spectroscopy, atomic force microscopy (AFM), and scanning electron microscopy (SEM) we elucidate the early stage formation of graphene by monitoring the transition from disordered carbon adsorbed on Cu to graphene. We also demonstrate for the first time (1) high‐throughput production, (2) ultralow cost, and (3) high quality monolayer graphene grown on Cu foils by resistively heated stage cold‐wall CVD. Our technique merges short deposition time (approximately few minutes) with high‐efficiency heating of a cold‐wall CVD system, resulting in ≈99% reduction in graphene production cost. The Raman spectra of our graphene films shows a low defect related peak and in devices with an area of 5600 μm^2^ fabricated on standard SiO_2_ substrates we measure a charge carrier mobility of 3300 cm^2^ V^−1^ s^−1^ and the quantum Hall Effect typical of single layer graphene. In contrast, the quality of graphene grown by hot‐wall CVD is often gauged only by carrier mobility,^1^, ^2^, ^4^, ^5^, ^21^, ^22^, ^23^ giving little information regarding the large area properties of the film. Therefore, to better quantify the quality of graphene films for electronic applications, we introduce an electronic quality factor (Q) accounting for the area across which the carrier mobility is measured. Using Q as a gauge we show that graphene grown by cold‐wall CVD has enhanced quality compared to the material grown by hot‐wall CVD. Finally, we demonstrate that graphene grown by cold‐wall CVD is suitable for the next generation electronics by embedding it into the first transparent and flexible graphene capacitive touch‐sensor that could enable the development of artificial skin for robots.

Studies of the growth mechanism of graphene on copper (using methane) in a hot‐wall CVD have thus far suggested the direct growth of 2D films involving several steps. The first step is the direct formation of 2D nuclei of graphene^24^ from the adsorbed carbon species resulted from the catalytic decompo­sition of methane on the copper surface. These graphene nucleation sites subsequently grow with the addition of carbon to their edges to form islands and large domains.^25^ The growth parameters such as the temperature, pressure, growth time, and gas flow are tuned to let graphene domains grow until they coalesce and a continuous graphene film is attained.^26^ Though it has been suggested that after the growth of the first layer the catalytic copper surface becomes passivated and limits the growth of other layers, several studies of low‐pressure CVD have reported the growth of bilayer^27^ and trilayer,^28^ as well as multilayers for atmospheric pressure CVD.^29^ Nevertheless, the thickness of the grown layers in a hot‐wall CVD is always limited to few nanometers or less.

Our experiments show that the growth mechanism of graphene in cold‐wall CVD is markedly different from that of the hot‐wall CVD described above. Specifically, over a range of growth temperatures that we have investigated, we always observed a thick carbon film (100 nm), which forms in the early stages of the growth (see **Figure** **1**a, top left), that becomes progressively thinner with increasing the growth time (see top inset in Figure 1b) and finally evolves into graphene islands (see Figure 1a, top right). The time required to form graphene decreases from 6 min at 950 °C to 20 s at 1035 °C (see the Supporting Information). To elucidate the initial stage of graphene growth, that is the adsorption of carbon on the Cu substrate, we focus on the slow graphene formation at 950 °C. Graphene films were obtained using a commercial cold‐wall CVD system (see the Supporting Information for details on the design and stability of critical parameters needed for the growth of high quality graphene with this process). The films were transferred from the Cu foils to SiO_2_/Si substrates using a wet transfer method.^1^, ^30^ Full details of the growth and transfer procedures are provided in the Supporting Information. Similar studies for films grown at higher temperatures are presented in the Supporting Information.

**Figure 1:**
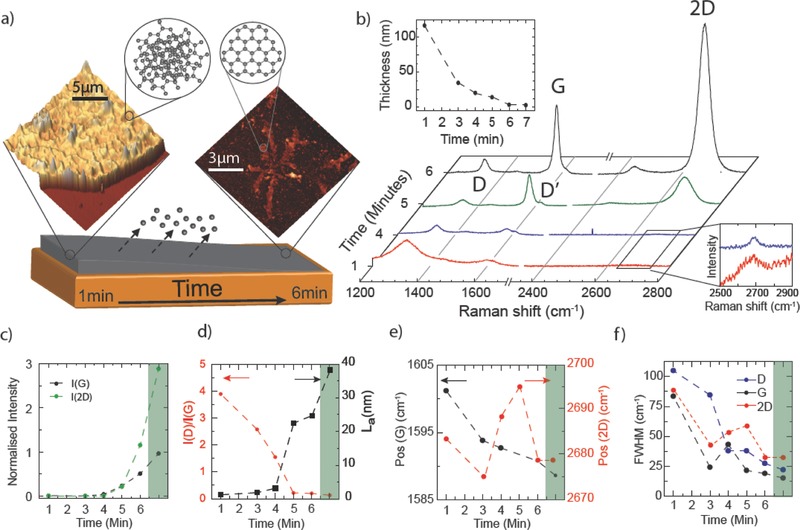
a) An illustration of graphene formation from a disordered carbon film. The inset images are AFM topographies for a disordered carbon film (left) and a graphene island (right). The top insets show schematic crystal structures of disordered carbon (left) and graphene (right). b) Raman spectra of films grown at 950 °C for different growth times and transferred to SiO2/Si. The peaks are normalized to the Si‐peak intensity. The top inset shows the film thickness as a function of growth time measured using AFM. The bottom inset shows the presence of 2D bands for *t*_G_ < 4 min. c) The intensity of the G‐ and 2D‐peaks normalized to the Si‐peak intensity as a function of growth time. d) The ratio of D‐peak intensity to G‐peak intensity and the sp2 cluster size (La) plotted as a function of growth time. e) It shows the evolution in G‐ and 2D‐ peaks position as a function of growth time. f) It shows the reduction in the FWHM of the D‐ and G‐ and 2D peaks as a function of growth time. The green regions in (c) to (f) indicate values taken from a continuous monolayer graphene film.

Figure 1b shows the Raman spectra of films grown at 950 °C for growth time (*t*_G_) ranging from 1 to 6 min. For all the samples we observe the characteristic peaks of sp^2^ bonded carbon atoms: the D‐peak at ≈1340 cm^−1^, the G‐peak around 1600 cm^−1^, the D′‐peak around 1620 cm^−1^, and the 2D‐peak at ≈2700 cm^−1^. For short *t*_G_ (i.e., 1–4 min) the D‐ and G‐peaks have considerable higher intensities than the 2D‐peak, which is typical of disordered carbon films.^31^, ^32^ As *t*_G_ increases we observe changes in intensities, sharpness, and positions of the D and G peaks, and for *t*_G_ > 4 min a well‐defined 2D‐band emerges. At the same time, AFM measurements show a reduction in the film thickness from 116 to 2.7 nm with increasing *t*_G_ from 1 to 6 min (see top inset of Figure 1b), which suggests the desorption of carbon from the film.

Lorentzian fitting of the D‐, G‐, and 2D‐peaks allows us to ascertain the structural ordering within the films by analyzing the band intensities (*I*_D,G,2D_), the full width at half maximum (FWHM(D,G,2D)) and the peak positions (Pos(G,2D)). According to the three stage model for classification of disorder,^33^, ^34^, ^35^, ^36^, ^37^ the evolution of *I*_D_/*I*_G_, FWHM(D,G) and Pos(G) allow us to assess the ordering/amorphization in carbon materials ranging from graphite and amorphous carbon^33^, ^34^ to few‐layer and monolayer graphene.^35^, ^36^, ^37^ For *t*_G_ = 1 min, the presence of a 2D peak with Pos(2D) = 2683 cm^−1^ and FWHM(2D) = 88 cm^−1^, the absence of a doublet in the D and 2D peaks, together with the overlap of G and D′ peaks indicate the formation of nanocrystalline graphite with no 3D ordering. Figure 1c shows that *I*_2D_ and *I*_G_ increase with increasing *t*_G_, whereas the ratio *I*_D_/*I*_G_ decreases from ≈3.9 to 0.2 (see Figure 1d). At the same time Pos(G) down‐shifts from 1601 to 1590 cm^−1^ (see Figure 1e) and a significant reduction of FWHM(D,G) occurs (see Figure 1f). The evolution of *I*_G,2D_, *I*_D_/*I*_G_, Pos(G), and FWHM(D,G) with increasing *t*_G_ is consistent with the stage 1 ordering trajectory leading from nanocrystalline graphite to graphite. In this regime the size of sp^2^ clusters (L_a_) increases with increasing ordering and can be estimated using the Tuinstra–Koenig relation *I*_D_/*I*_G_ = C(*λ*)/L_a_ where C(532 nm) ≈ 4.96 nm.^38^, ^39^ Using this relation we estimate L_a_ ≈ 2 nm for *t*_G_ = 1 min, which increases to L_a_ ≈ 25 nm for *t*_G_ = 6 min as shown in Figure 1d.

For *t*_G_ > 6 min the 2D‐peak intensity is larger than two times the intensity of the G‐peak and it can be fitted with a single Lorentzian, with Pos(2D) = 2678 cm^−1^ and FWHM(2D) = 30 cm^−1^ indicating the formation of monolayer graphene.^35^, ^40^, ^41^ This conclusion is supported by AFM measurements showing the formation of islands with a thickness of 2.7 nm, which corresponds to monolayer graphene and accounts for fabrication residues and substrate effects.^42^ Furthermore, electrical transport measurements performed on continuous films with a similar Raman spectra and AFM thickness show the quantum Hall effect typical of monolayer graphene as discussed later.

To provide further insights into the transition from nanocrystalline graphite film to graphene islands we monitor the evolution of the density, size, and separation of the islands using SEM observations combined with a simple counting algorithm described in the Supporting Information. **Figure** **2**a shows the evolution from a continuous film to discrete islands with increasing growth time for 950 °C. These images have been performed on the same samples used for the Raman measurements in Figure 1. The average island area within the same range of growth times is shown in Figure 2b, whereas the average separation between islands at initial fragmentation then from 4 to 10 min is shown in Figure 2c. An initial reduction in island size suggests desorption of material from the surface. The observed saturation in the island separation of 7.23 μm indicates that there is no further nucleation of islands after the initial fragmentation. After 7 min we see a maximum in island size of 19.7 μm^2^. Raman measurements confirm that these islands are composed of graphene. SEM analysis of films grown for 1000 and 1035 °C reveals a similar behavior of the saturation in island separation and a maxima in island size (see the Supporting Information). We observe that an increase in growth temperature leads to a reduction in the time required to achieve the maximum island size and to form a monolayer graphene as shown in the inset of Figure 2c. A similar behavior has been also observed in other CVD graphene growth studies,^24^, ^26^ which showed that the growth rates of graphene islands are determined by competing atomic phenomena such as adatom mobility and attachment to the islands edges versus desorption, as well as being affected by the microscopic substrate roughness.^26^ The counterintuitive decrease in island area with time can be understood within the desorption controlled regime^26^ where the growth is a thermally activated process with a barrier energy *E*_a_ = (*E*_des_ + *E*_att_ – *E*_d_ – *E*_ad_)/2 and with the density of graphene islands N_i_ ≈ PCH_4_· exp(2*E*_a_/KT), with *E*_des_ the desorption energy of a carbon monomer on the Cu surface, *E*_att_ the barrier of attachment for the capture of a monomer by supercritical nucleus, *E*_d_ the activation energy of surface diffusion of a monomer, *E*_ad_ the activation energy for dissociative adsorption of CH_4_ on Cu, PCH_4_ the methane partial pressure, K the Boltzmann constant, and T the growth temperature. Figure 2d shows that when the island area decreases with time, N_i_ has a dependence on growth temperature which is typical of the desorption controlled regime with an activation energy of 1.66 eV. The desorption model is also consistent with the formation of holes inside the islands at 8 min of growth (see Figure 2a).

**Figure 2:**
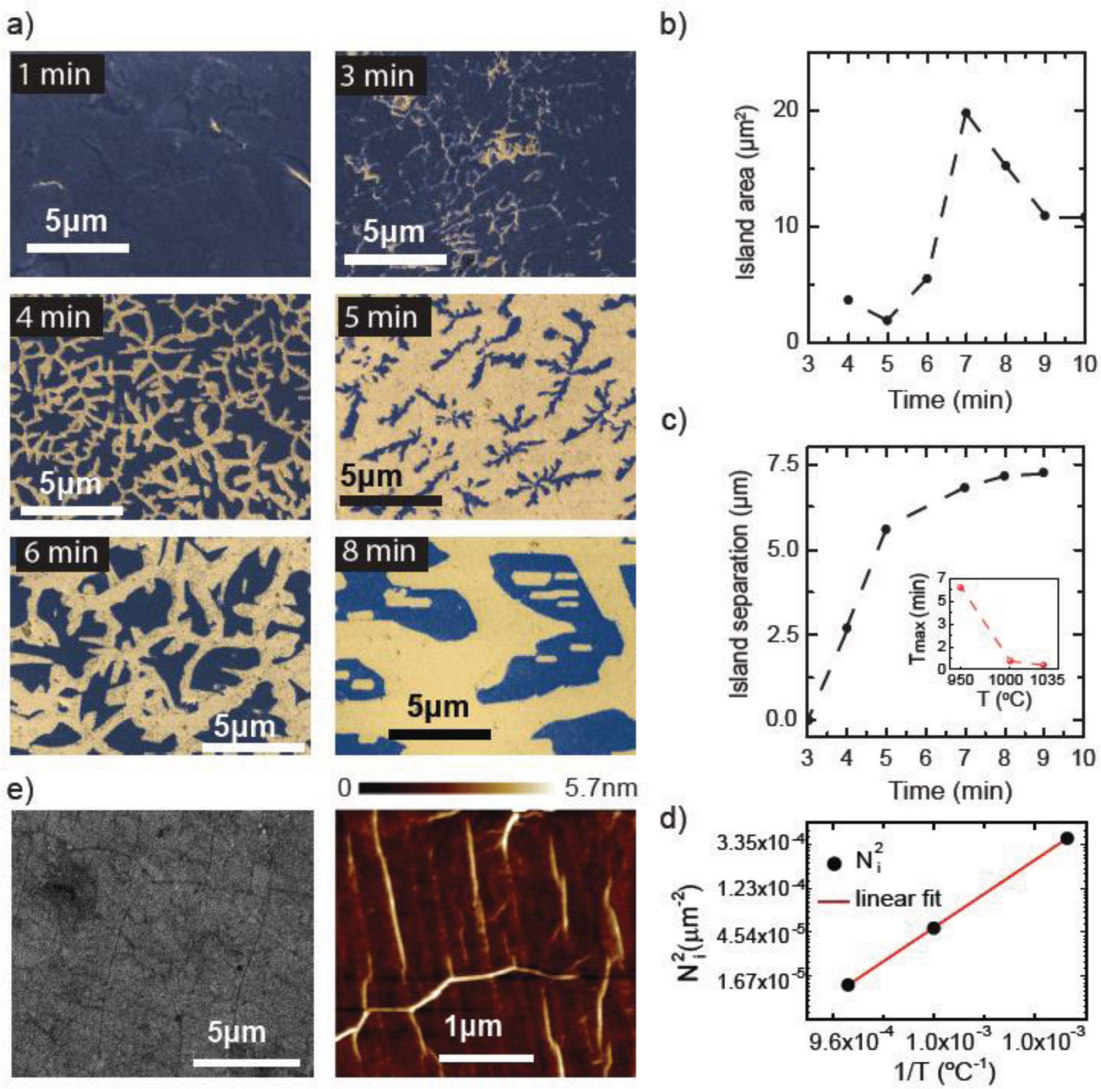
a) SEM images sampled over a time frame of the transition from nanocrystalline graphite to graphene. The dark blue color corresponds to graphene, whereas the yellow color is the substrate. b) The evolution of the average island area with the growth time. c) The average separation of islands as a function of growth time. The inset shows the time to reach maximum island size *T*_max_ plotted as a function of growth temperature. d) Graphene island density as a function of inverse of growth temperature in the regime where the island area decreases with growth time. The red line is a fit to the desorption controlled regime model. e) SEM (left) and AFM (right) images of the continuous monolayer graphene films.

The observed transition from a disordered carbon film adsorbed on Cu to graphene is very likely due to the combination of high temperature, low pressure, and the presence of the catalytically active surface of Cu, which induces the conversion to graphene as well as the thinning process of the carbon film. Previous studies^43^, ^44^, ^45^ have also investigated the high temperature conversion of amorphous carbon (a‐C) films into graphene. In situ transmission electron microscopy (TEM) and molecular dynamics (MD) studies^43^ have reported the high temperature conversion of amorphous carbon (a‐C) into graphene patches of 100 × 300 nm^2^. It was shown that a‐C can rearrange into graphene through a phase of glasslike carbon which takes place within a time frame from 1 to 15 min, in the temperature range of 326–926 °C. Another study^44^ showed that graphene can be grown in a solid‐state transformation of a‐C in the presence of a catalytically active metal at temperature up to 720 °C. In this case rearrangement processes take place in two or 3D unordered network structures in which a huge number of bonds are broken and newly formed. Finally, a third study showed the metal‐catalyzed crystallization of a‐C to graphene by thermal annealing at 650–950 °C.^45^ It was shown that part of the carbon source is crystallized into graphene with the rest outgassing from the system. Furthermore, this study also reports that for long annealing times no carbon or graphene remains on the surface due to significant desorption of C atoms under the low pressure and high temperature ambient. Similarly to these studies we have a film of nanocrystalline graphite on top of a catalytically active metal in low pressure and high temperature conditions, as well as comparable time frames for the conversion to graphene.

Having established the initial stages of graphene formation, we investigate the transition from graphene islands to a continuous film. Figure 2c shows that the island size reaches a maximum with the growth time and a further increase in the growth time leads to a decrease in the island size. To grow continuous graphene monolayer films we adopted the two stage growth described by Li et al.,^26^ where increasing methane flow rate after the formation of the islands is shown to fill the regions between islands while suppressing further nucleation sites. As our objective is to minimize growth time, we selected the growth temperature of 1000 °C where maximum island size and island separation are reached in the shortest time (40 s). Using the grown graphene islands as nucleation sites, we find that increasing the methane flow rate and growth time to 5 min allows the islands to merge into a continuous graphene monolayer film of up to 8 cm^2^ in area. SEM, AFM, and Raman measurements confirm that the continuous films are monolayer graphene. Figure 2e shows the morphology of the graphene monolayer after the complete coalescence of the islands studied by SEM and AFM. The analysis of the Raman measurements performed on the continuous films is presented in Figure 1, where the green highlighted regions in panels (c) to (f) indicate the values of *I*_G,2D_, *I*_D_/*I*_G_, L_a_, Pos(G), and FWHM(D,G,2D) for a 1 × 1 cm graphene film. Raman mapping measurements shown in the Supporting Information demonstrate the uniformity and high‐quality of the continuous films. The total processing time of this procedure is about 20 min (see the Supporting Information); this includes (1) heating up time for the CVD system from room temperature to the growth temperature, (2) Cu foil annealing time, (3) graphene nucleation and growth time, (4) cooling down time for the system to room temperature. The demonstrated processing time is significantly shorter than the processing time needed by hot‐wall CVD (typically >70 min).^1^, ^2^, ^4^, ^5^, ^21^, ^22^ We estimate the total cost of graphene production by cold‐wall CVD to be <0.37 cm^−2^ (see the Supporting Information). Compared with other CVD studies and neglecting the base cost of copper we see a reduction in the production costs of 98.83%–99.89%. This extraordinary reduction in the production cost, together with the possibility of reconstitution of high purity copper from etchant solutions by electrolysis that can yield up to 99% of the original foil,^46^ open a new way forward to accelerate the commercialization of graphene.

To ascertain the quality of the electronic properties of graphene produced by cold‐wall CVD we characterized the charge carrier mobility in transistor devices fabricated on SiO_2_/Si substrates. Using the parallel plate capacitor model we estimate the field effect mobility to be 3300 cm^2^ V^−1^ s^−1^ at 1.4 K and 2773 cm^2^ V^−1^ s^−1^ at room temperature. This mobility, measured across a large area device (≈0.05 mm^2^), is comparable to the mobility measured in smaller area devices of graphene either grown by CVD (50 μm^2^ 1 to 0.03 mm^2^ 23 or mechanically exfoliated (typically few μm^2^) and deposited on oxidized silicon substrates.^1^, ^2^, ^4^, ^5^, ^21^, ^22^, ^47^, ^48^, ^49^ The quality of cold‐wall CVD graphene as compared to that grown with other methods is readily assessed using the electronic quality factor (Q) that, for the area across which the carrier mobility is measured, is defined as the field effect mobility (cm^2^ V^−1^ s^−1^) multiplied by the area of the device (μm^2^). As shown in the Supporting Information, graphene grown by resistive heating cold‐wall CVD has Q ranging from 4 × 10^6^ to 7.2 × 10^6^, whereas most reports of monolayer graphene grown by hot‐wall CVD have Q ranging from 10^3^ to 7 × 10^6^. Hence cold‐wall CVD grown graphene has a narrow spread of Q stemming from a reproducible high quality growth process. This is in stark contrast to the spread of Q over three orders of magnitude reported for hot‐wall CVD grown graphene.

**Figure** **3**a shows the four‐terminal resistance measured in a large Hall bar geometry (225 × 25 μm^2^, see inset) fabricated on standard SiO_2_/p‐Si substrates. The Si substrates are heavily doped and act as the gate electrode. By applying a voltage to the gate (*V*_g_) we tune the carrier concentration from 3 × 10^11^ to 6 × 10^12^ cm^−2^. The charge neutrality point is at 0 V, indicating low residual doping levels in our samples. Figure 3b shows the resistivity and the Hall conductance (*σ*_xy_) against the applied *V*_g_, taken at 13 T and at a temperature of 250 mK. Clearly developed conductance plateaus are visible when the Fermi energy is within a Landau level (N) that correspond to the conductance relationship *σ*_xy_ = (N + 1/2)4e^2^/h, typical of the half‐integer quantum Hall effect (QHE) of monolayer graphene with two‐fold spin and valley degeneracy.^38^, ^49^ At the same time we observe *ρ*_xx_ = 0 Ω where the Fermi energy is within the N = 0 Landau level for both electrons and holes, indicating that the graphene quality is high enough to observe the localization in the QH regime. At the same time, *σ*_xx_ shows well‐defined Shubnikov–de Haas oscillations which are periodic with the applied gate voltage. A color map of the differential Hall conductance plotted against perpendicular applied field and charge carrier concentration shows that Landau levels up to N = 6 are visible at high fields (see Figure 3c). The N = 1 Landau level is visible down to fields as low as 5 T. The presence of these QHE features at low fields is an indication of low disorder in the graphene which further demonstrates the high electronic quality of cold‐wall CVD graphene.

**Figure 3:**
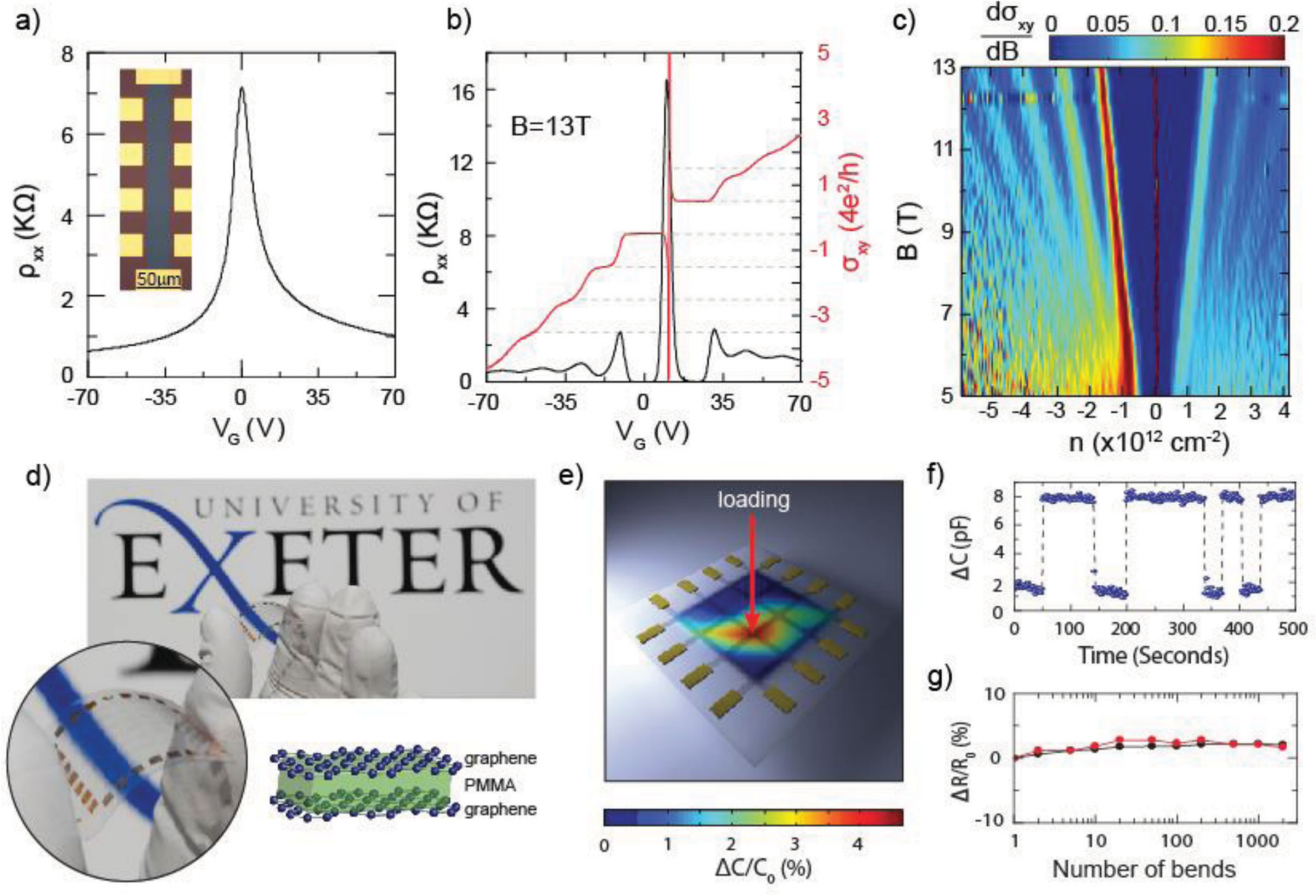
a) The longitudinal resistivity (*ρ*_xx_) plotted against applied gate voltage at 4.2 K. The inset shows a false color photograph of the device. b) The longitudinal resistivity and the Hall conductance (*σ*_xy_ normalized to 4e^2^/h) plotted against applied gate voltage at 250 mK with a 13 T perpendicular applied field. c) Color map of the differential conductance as a function of applied perpendicular magnetic field and carrier density. d) Shows a photograph of a flexible and transparent graphene touch sensor and a schematic of the touch sensor device. e) Color map of the change in capacitance when a single element is loaded with a 36 g mass. f) The change in capacitance of one element with respect to time when pressed with a human finger. g) The change in line resistance after flexing the device about a 2.5 cm radius. Black and red points show the resistance of line parallel and perpendicular to the bending radius, respectively.

In the final section of this article we demonstrate that graphene produced by this novel method is suitable for the next generation flexible and transparent electronics. In such applications touch sensing is the dominant human interface method for detecting an input. Among the touch sensing devices, the capacitive touch sensors have the fastest response time and the best sensitivity to touch input. However, graphene‐based flexible capacitive touch sensors have not been demonstrated so far due to the difficulties arising from poor adhesion of subsequent graphene and dielectric layers on flexible substrates. We developed a novel fabrication procedure that preserves the high quality of graphene (see the Supporting Information), therefore allowing us to demonstrate for the first time a flexible and transparent capacitive touch sensor using graphene for both the top and bottom electrodes.

Figure 3d shows a photograph of a capacitive touch sensor array fabricated on a flexible and transparent polyethylene naphthalate (PEN) substrate. The array consists of two orthogonal sets of graphene strips separated by poly(methyl methacrylate) (PMMA) dielectric as illustrated in Figure 3d. The individual graphene strips were fabricated on the Cu foil and connected to 50 nm thick Au pads, followed by their transfer to the PEN substrate. Electrical transport measurements show that the typical resistivity across each strip length is *ρ* ≈ 1.3 kΩ and the contact resistance <68 Ω. A detailed description of the fabrication procedure and electrical characterization is provided in the Supporting Information. The individual elements of the touch sensors are formed at the intersection between the graphene strips and each element represents a parallel plate capacitor. As pressure is applied to an element of the array, the dielectric elastically deforms reducing the spacing between the graphene electrodes resulting in an increase in capacitance. Figure 3e shows an interpolated color map of percentage change in capacitance for each element when one element is loaded with a 36 g mass. The maximum change in capacitance occurs on the loaded element with minimal changes to the surrounding elements.

To test the responsivity of the device we periodically loaded and unloaded an element with a human finger and measured the change in capacitance shown in Figure 3f. A change in capacitance during loading of Δ*C* = 6 pF was observed with a return to the original state after unloading. The sharp change in capacitance demonstrated indicates a fast responsivity to loading and unloading of the element. Finally we tested the flexibility and durability of the devices by bending the substrate and systematically measuring the resistance of the graphene strips across the device. Figure 3g shows the percentage change in the two terminal resistance of the graphene strips as the device is flexed though a 2.5 cm bending radius for 2000 iterations. This test was performed for graphene strips parallel (black) and perpendicular (red) to the axis of device flexing. After 2000 bends only minor changes of less than <2.7% in the line resistance are observed, which show no significant deterioration of the operation of the flexible touch sensor. These measurements demonstrate the sensitivity and durability of our graphene touch sensor, and its suitability for use in next‐generation flexible portable devices.

In summary, we have shown a new growth mechanism of graphene by cold‐wall CVD, which starts with the formation of a thick carbon film in the early stages of the growth, that becomes progressively thinner with increasing the growth time and finally evolves into graphene islands. At the same time we demonstrate an extremely high‐throughput and cost efficient growth procedure for preparing high quality monolayer graphene using cold‐wall CVD. Finally, we use graphene as electrode material and demonstrate the first flexible and transparent graphene capacitive touch sensor using processing techniques that are compatible with existing transparent and flexible electronic technologies. Besides its importance for the quick industrial exploitation of graphene since cold‐wall CVD systems are found in semiconductor industries manufacturing plants, our work could lead to new generations of flexible electronics and offers exciting new opportunities for the realization of graphene‐based disruptive technologies.

## Supporting information

As a service to our authors and readers, this journal provides supporting information supplied by the authors. Such materials are peer reviewed and may be re‐organized for online delivery, but are not copy‐edited or typeset. Technical support issues arising from supporting information (other than missing files) should be addressed to the authors.

Supplementary
