# Supplementary material for: High Quality Monolayer Graphene Synthesized by Resistive Heating Cold Wall Chemical Vapor Deposition
Source: Adv Mater. 2015 Jun 5;27(28):4200–6. doi: 10.1002/adma.201501600 (PMC4744682; doi:10.1002/adma.201501600)
Supplement: Supplementary file 1 — Supplementary [file ADMA-27-4200-s001.pdf]

Copyright WILEY-VCH Verlag GmbH & Co. KGaA, 69469 Weinheim, Germany, 2015.

ADVANCED MATERIALS

Supporting Information

for *Adv. Mater.,* DOI: 10.1002/adma.201501600

High Quality Monolayer Graphene Synthesized by Resistive Heating Cold Wall Chemical Vapor Deposition

*Thomas H. Bointon, Matthew D. Barnes, Saverio Russo, and Monica F. Craciun**

Copyright WILEY-VCH Verlag GmbH & Co. KGaA, 69469 Weinheim, Germany, 2013.

Supporting Information

High quality monolayer graphene synthesized by resistive heating cold wall chemical vapour deposition

*Thomas H. Bointon, Matthew D. Barnes, Saverio Russo, and Monica F. Craciun**

Description of the cold-wall CVD system used for the growth of graphene

The growth of graphene was performed in a commercial cold-wall CVD system from Moorfield (i.e. nanoCVD-8G system). In the following we provide a description of the system as well as the required parameters and schematic representations needed to grow graphene with any resistive heating cold-wall CVD process.

This system is enclosed in a metallic case and consists of a stainless steel vacuum chamber, mass flow controllers for the gas delivery, valves for chamber pressure control and purge, and a gauge for pressure measurement (see Figure S1). The system is equipped with 3 types of process gasses (CH4, H2 and Ar). However in this study we only used CH4 and H2 for the growth. The purge gas is argon.

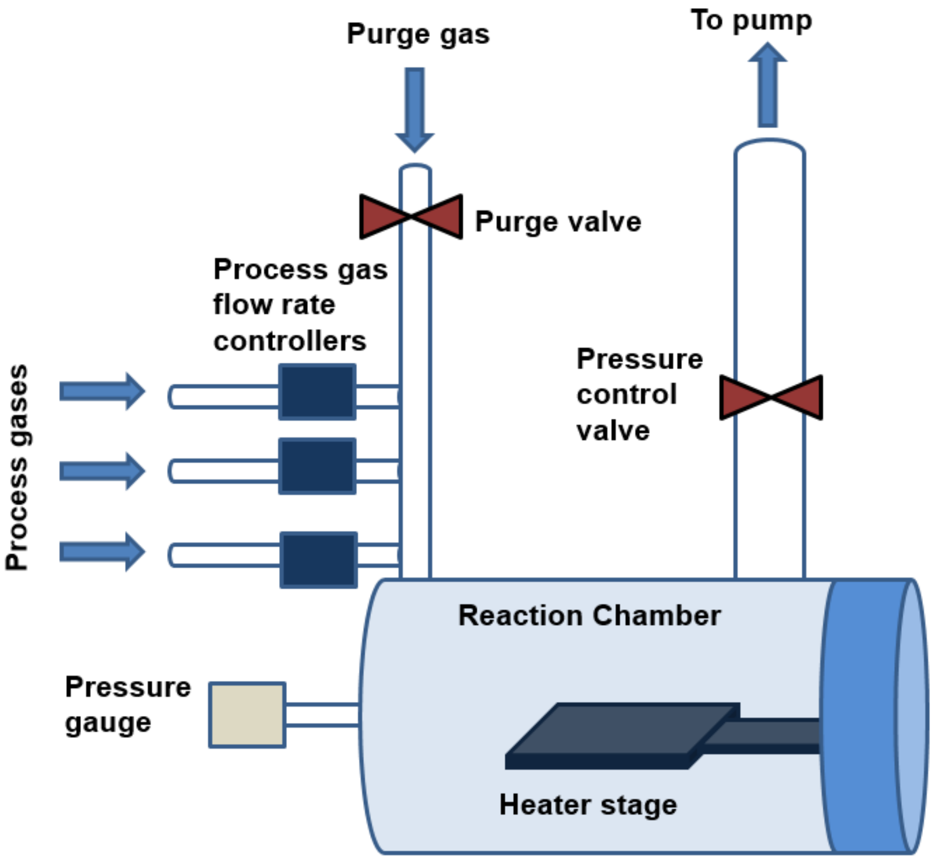

Figure S1: Schematic diagram of the cold-wall CVD system used for graphene growth. The arrows indicate the direction of gas flow.

The reaction chamber houses a resistively heated substrate stage equipped with an embedded thermocouple which can achieve stable temperatures of up to 1100°C. The heater assembly slides out of the chamber for substrate loading (see Figure S2) and is then pushed back in the chamber. The hardware is controlled by a programmable logic controller electronics coupled to a touchscreen interface and all operation of the system is carried out through the touch screen. In this system the Cu foil is placed on the resistively heated stage as shown in Figure S3.

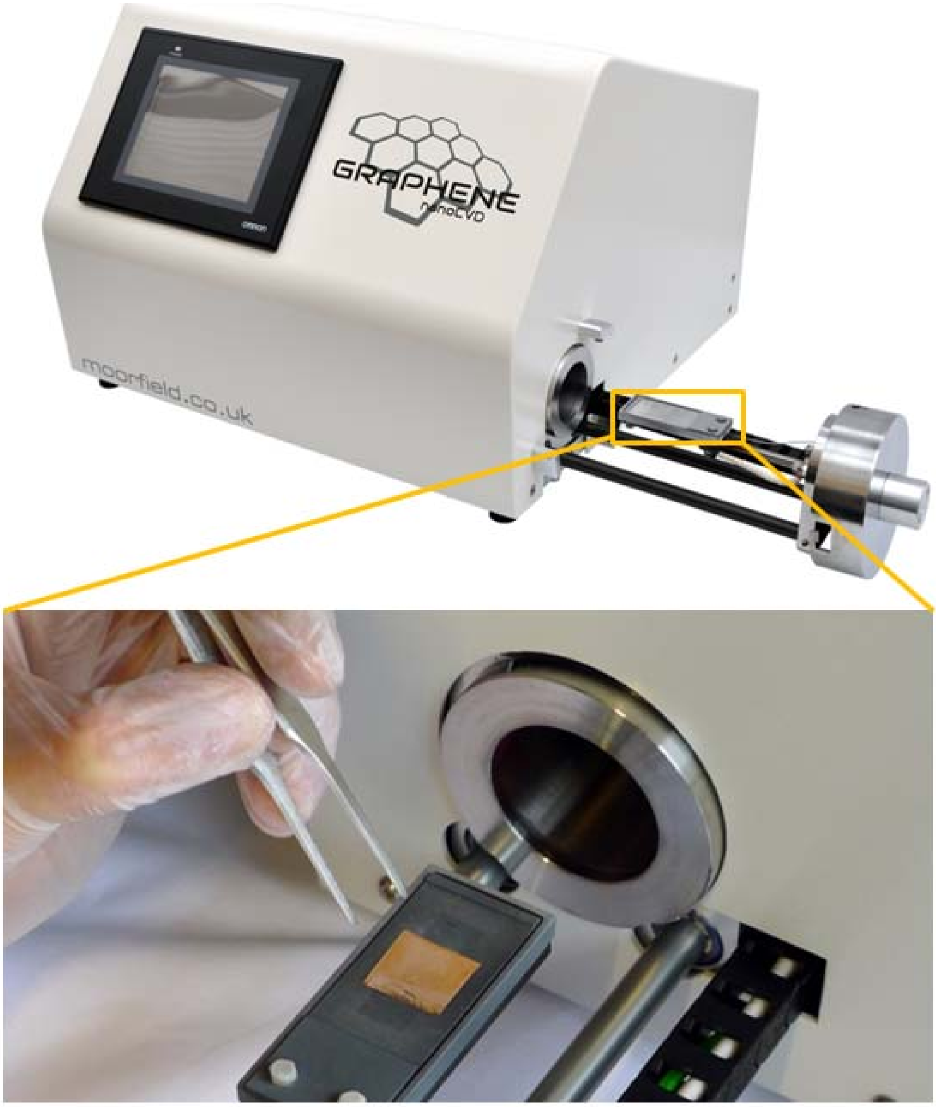

Figure S2: A photograph of the CVD system used in our work. The inset shows the resistive element heating stage loaded with a Cu foil [reproduced with permission from the website of Moorfield nanoCVD-8G].

The temperature at the surface of the Cu foil is measured by using a thermocouple mounted on the heater stage, thus in direct contact with the substrate. Figure S11a shows the heater stability for different temperatures as well as the chamber temperature which remains around 100°C during the heater operation. As the Cu foil is in direct contact with the heater/thermocouple, the temperature of the substrate can be reliably controlled as the introduction of gas does not modify the foils surface temperature (see Figure S3b).

a)

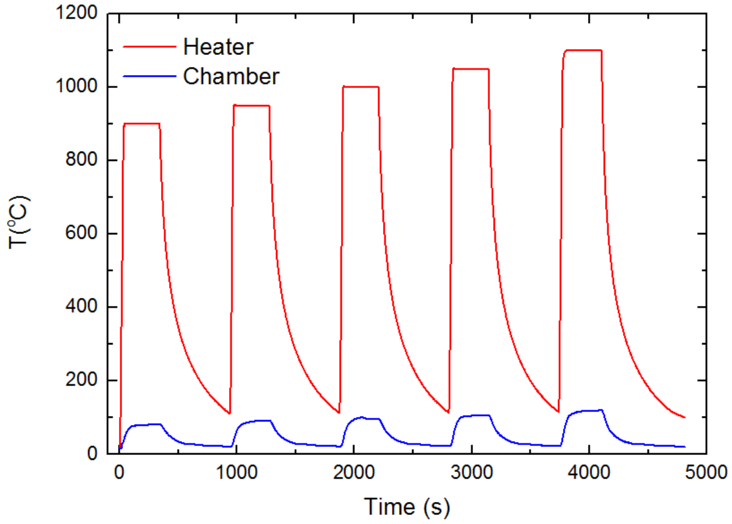

b)

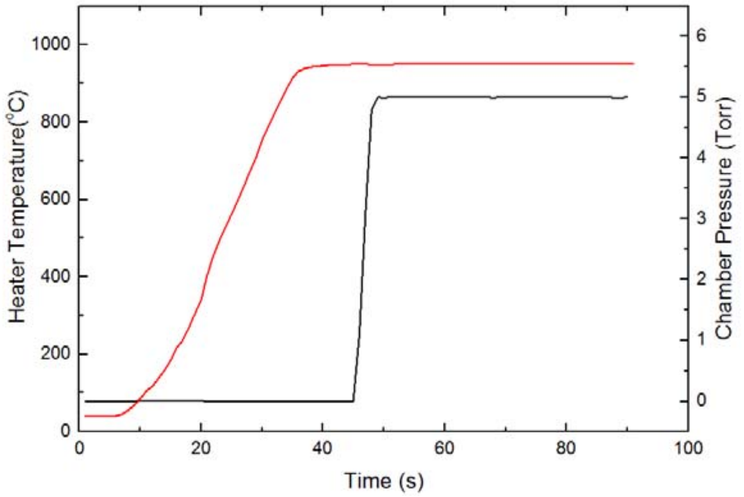

Figure S3: a) The stability of the heater temperature (red) in vacuum (P=0.05 Torr) for different temperature set-points ranging from 900°C to 1100°C. The blue curves show the corresponding chamber temperature which is around 100°C. b) The stability of the heater when gas with a pressure of 5Torr is introduced in the system.

The pressure inside the reaction chamber can be reliably controlled using the pressure control valve. Figure S4 shows the pressure stability for different set-points which are achieved in this case by controlling the flow of Ar gas.

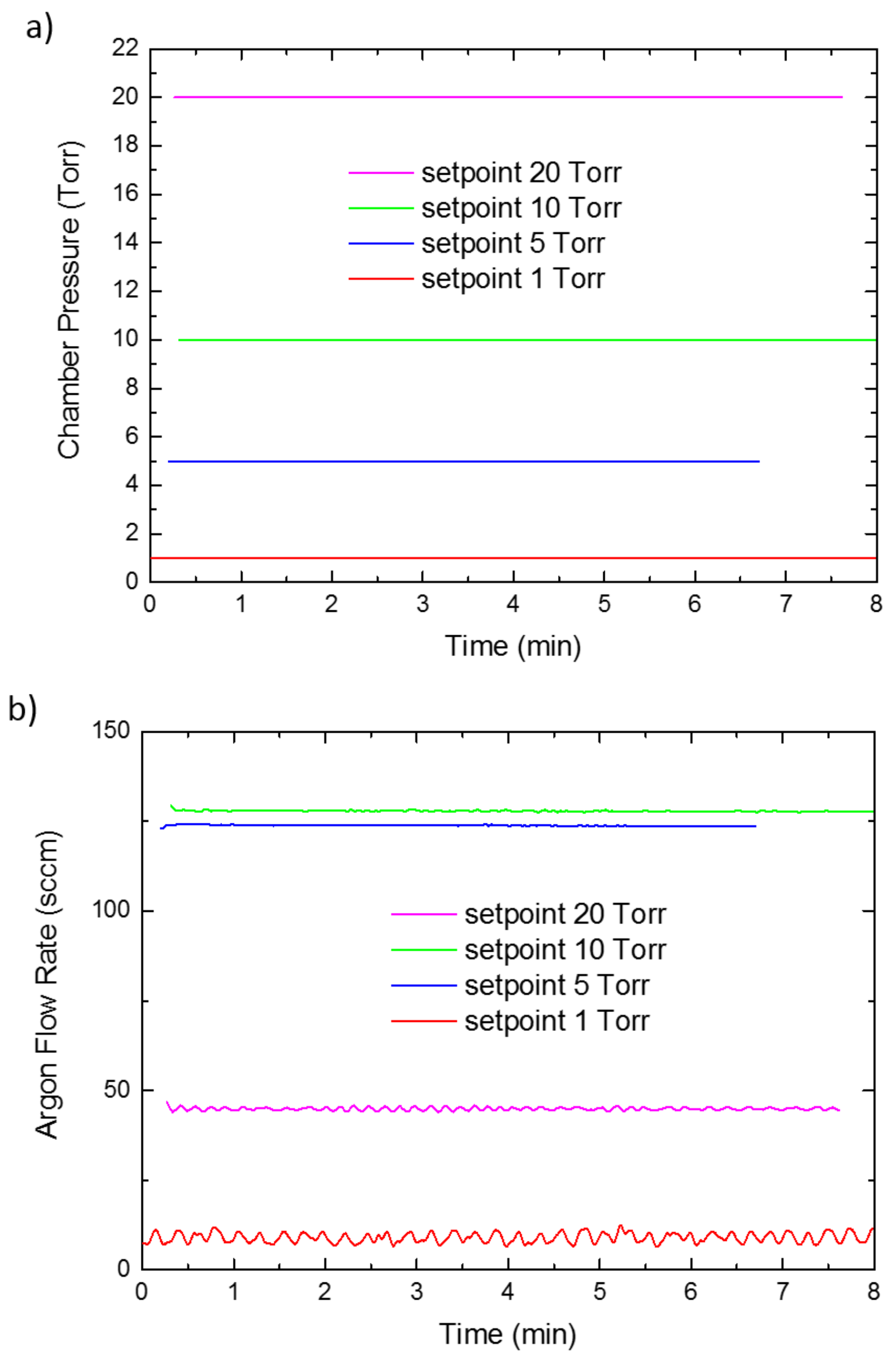

Figure S4: Pressure stability for different set-points (a) and the gas flow required to achieve the desired pressure (b).

Growth procedure for the graphene films and islands

25 μm thick copper foils (Alfa Aesar 99.999%) were annealed for 10 minutes at 1035°C in a H_2 atmosphere to increase the Cu grain size.

To understand the initial stages of graphene formation, the growth was carried out at temperatures ranging from 950°C to 1035°C and the growth time was varied from 10 seconds to 600 seconds. A constant flow rate of 0.4sccm of H_2 and 1.4sccm of CH_4 was used for all growths.

A typical processing for the growth of continuous graphene films involves the following steps: (1) heating up the CVD system from room temperature to the growth temperature, (2) Cu foil annealing, (3) graphene nucleation and growth, (4) cooling down the system to room temperature (see Figure S5).

During the heating up stage H_2 gas was flown at a rate of 0.4sccm with a chamber pressure of 0.01 Torr. The annealing step was performed for 10 minutes at 1035°C in a H_2 atmosphere, keeping the H_2 gas flow rate at 0.4sccm and the chamber pressure of 0.01 Torr. The temperature was then lowered at 1000°C for the growth of continuous graphene films. A constant flow rate of 0.4sccm of H_2 was kept throughout the nucleation and growth. For the nucleation stage, 1.4sccm of CH_4 was introduced for 40 seconds. This was followed by the growth stage where the CH_4 flow rate was increased to 7sccm for a 300 seconds. Finally, the system was cooled down at room temperature keeping the H_2 gas flow rate at 0.4sccm.

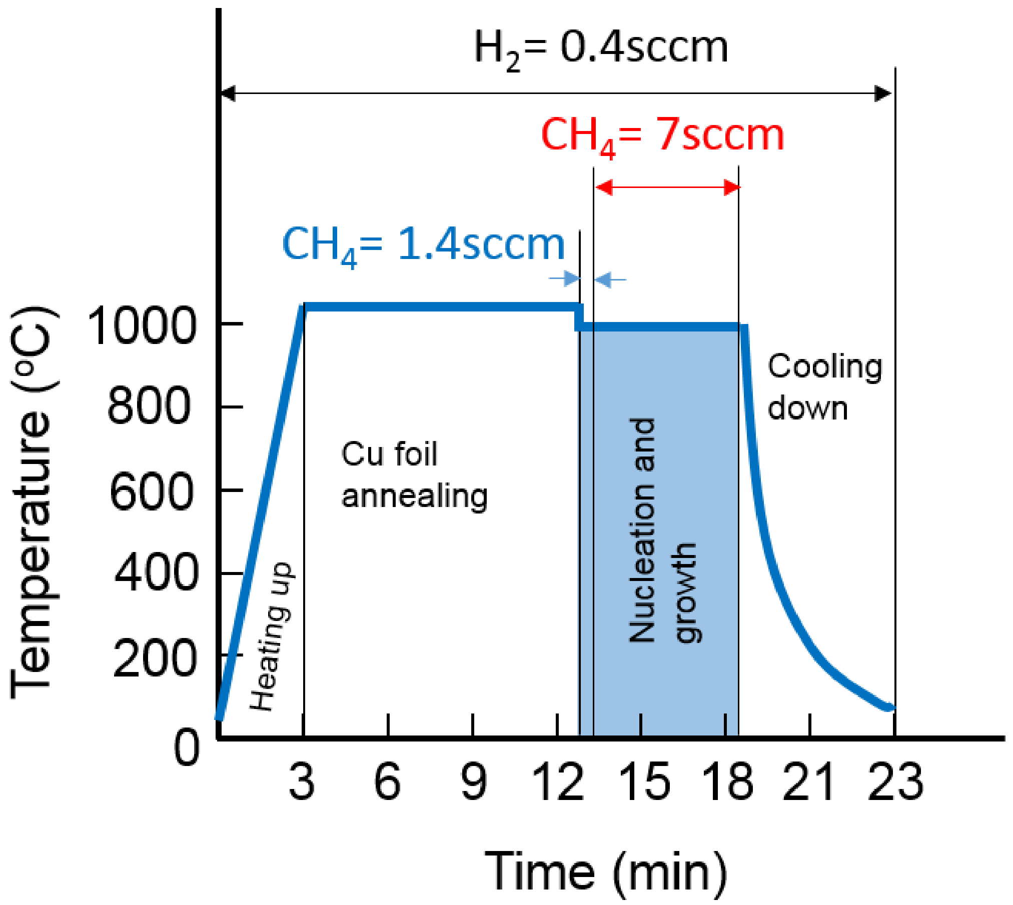

Fig. S5. Time dependence of growth parameters for the cold-wall CVD system used in this study.

Transfer Procedure of graphene films from the Cu foils onto SiO_2/Si

Grown graphene samples were spun with 200nm of 950K PMMA.The PMMA coated foils were vacuum cured for 30 minutes and then etched in 1M FeCl3 solution. After the copper was fully etched the films were transferred several times to deionized water and then transferred onto SiO2/Si substrates.

Device fabrication

Graphene devices were produced using standard electron beam lithography and reactive ion etching techniques to define Hall bar geometry (225 μm × 25 μm) shown in the false colour inset of Figure 3a with electrical contacts of Au/Cr (50 nm/ 5 nm).

Electrical transport measurements

The longitudinal and Hall voltages were measured in a four terminal geometry applying an AC current using a lock-in amplifier. The excitation voltage was selected to be within the linear transport regime.

SEM analysis

SEM Measurements: SEM micro-graphs were collected with a Phillips SEM. An acceleration voltage of 30kV, magnification of x5000 and beam current of 0.63nA was used.
SEM micrographs where taken for graphene islands transferred to SiO_2 to determine the average area and separation of domains. Figure S6a shows a micrograph taken at 5000x magnification where graphene islands appear dark and the SiO_2 substrate is lighter. The image was then processed by inverting the colors and applying a threshold to create a two colour bitmap shown in Figure S6b. Using the matlab image processing toolbox, each island was identified and the area was measured [1]. Figure S6c demonstrates a single identified island on a false colour map. To reduce the effects of residues resulting from the transfer process the results were filtered to remove any island with an area smaller than 1 μm^2. The resulting islands were given random false colour to check that no islands are connected as demonstrated in Figure S6d.
All calculations were based on 10 micrographs for each growth time, where the average area was estimated by summing the area of all islands ($A_{islands}$) and dividing by the total number of islands ($N_{islands}$). The average separation was estimated from the density of islands (S_{mean})

$$S_{mean} = \sqrt{\frac{1}{d}} = \sqrt{\frac{A_{total}}{N_{islands}}}$$

where density (d) was taken as the total number of islands ($N_{islands}$) divided by the total area of the micrographs (A_{total}).

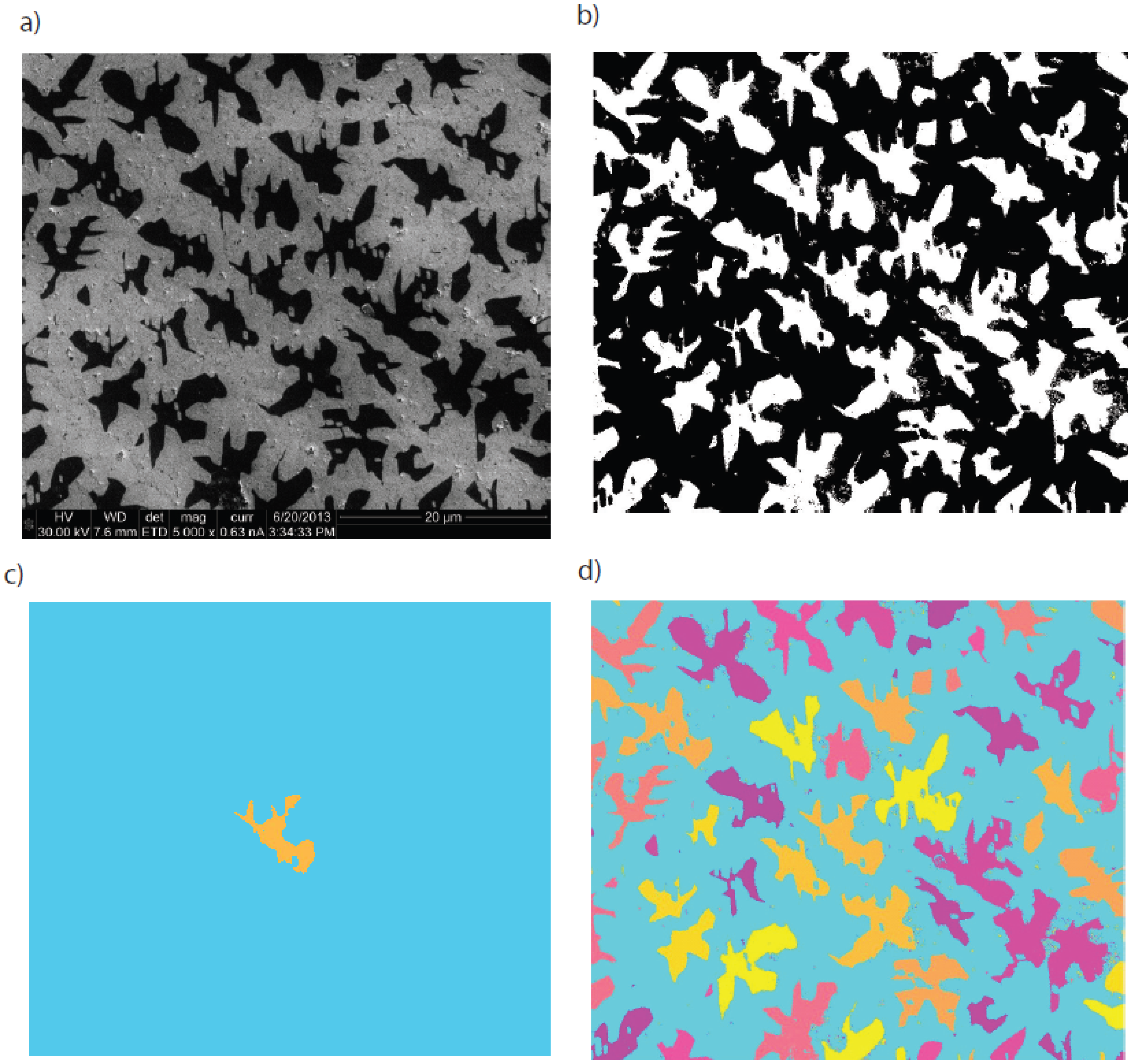

Figure S6. a) An SEM micrograph showing Graphene islands (Black) on an SiO_2 substrate, b) Processed SEM micrograph with inverted intensities and applied black and white threshold, c) A single identified island extracted from SEM micrograph shown in false colour, d) All identified islands from SEM micrograph after applying noise filter

AFM analysis

To study the evolution from a carbon film to graphene islands, semi-contact AFM topography images were collected with a NTMDT Ntegra AFM. Film thickness was extracted by fitting the statistical distribution of the film and substrate heights. For the contious graphene films, the images were colected in contact mode with a Bruker Innova AFM.

The thickness of each growth time was determined using tapping mode AFM where a surface topography was measured, shown in Fig. S7a. An area which includes the substrate and the film/islands highlighted in Fig. S7a was sampled and the distribution was then plotted as a histogram shown in Fig. S7b. The two peaks represent the substrate height and the film/island height. Fitting each peak with a Gaussian and subtracting the height of the substrate from that of the film/islands gives the total film thickness.

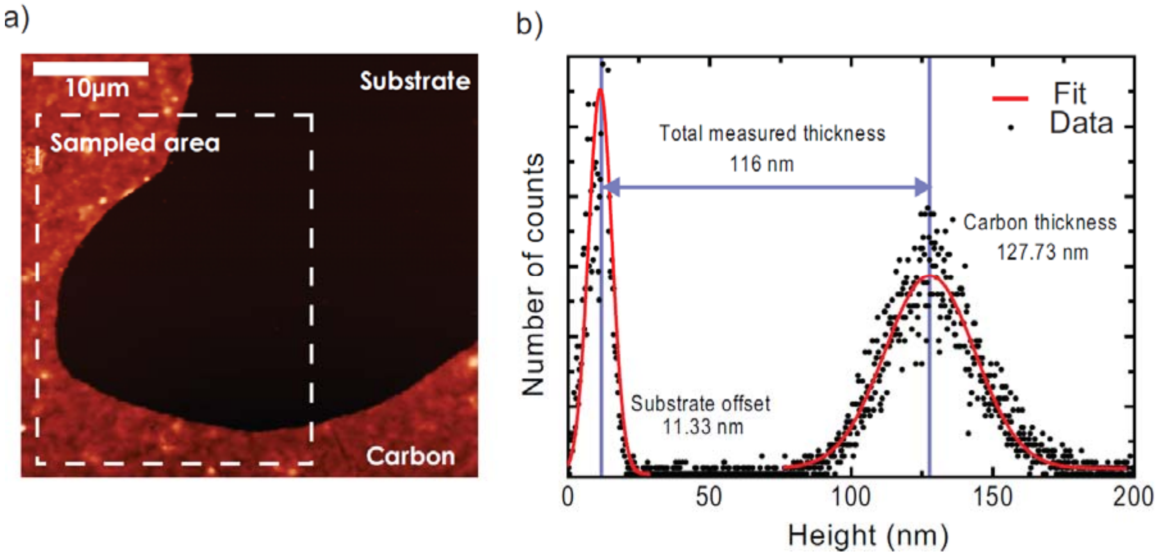

Figure S7. a) AFM topography of graphene film on a SiO_2 substrate, the highlighted region shows the sampled region for the statistical study. b) A histogram showing the distribution of measured heights from within the sampled area. Substrate and film distributions are fitted and the difference between the average heights gives the total thickness.

Raman spectra for films grown at 1000°C and 1035°C

Raman spectra were collected in a Renishaw spectrometer with an excitation laser wavelength of 532 nm, focused to a spot size of 5 µm diameter and x50 objective lens.

For films grown at higher temperatures (1000°C, 1035°C) we observe the same transition from nanocrystalline graphite to graphene islands as for growths at 950°C shown in Figure 1, but at a faster rate. Figure S8a shows several spectra at 1000°C for different times. After 5 seconds of growth we observe the presence of D- and G-bands alongside a small 2D-band characteristic of nanocrystalline graphite. As growth time is increased there is an increase in the intensity of the 2D-band and the ratio of the band intensities of D- and G-band, I_D/I_G transitions from ~2 to <1 demonstrating the increase in long range hexagonal ordering. Furthermore after 40 seconds $I_G/I_{2D} \approx 0.5$ indicating that the islands are monolayer graphene. Similarly Fig. S8b shows several spectra for growth at 1035°C for different times. We observe the spectra of nanocrystalline graphite after 1 second and a transition into monolayer graphene after 20 seconds.

There is a clear relationship between the growth temperature and the time for the nanocrystalline graphite to transition into monolayer graphene, where increasing the temperature reduces the time required to form graphene.

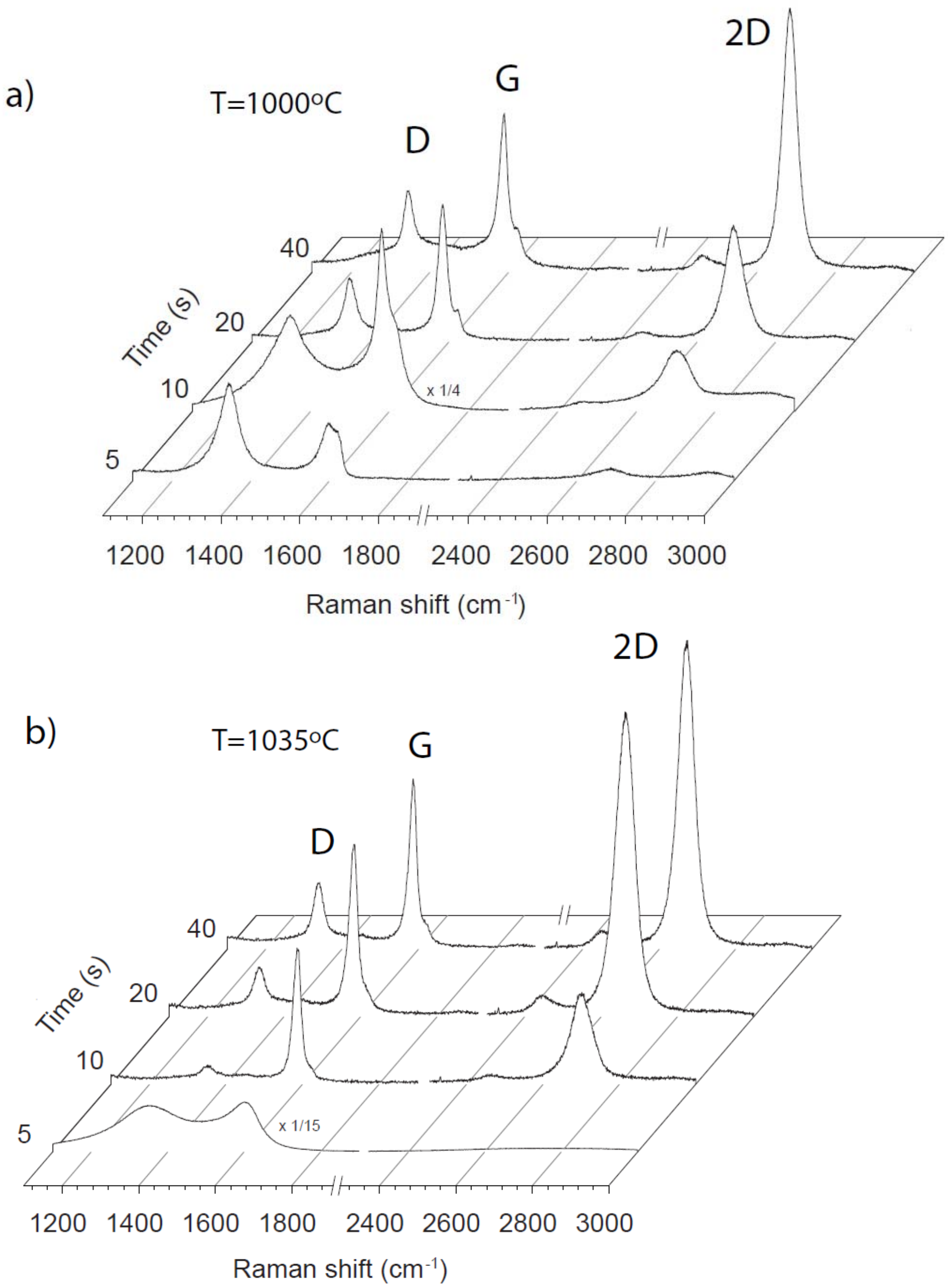

Figure S8 The evolution with respect to growth time of the Raman spectra for growths at a) 1000°C and b) 1035°C.

Island area and island separation for films grown at 1000°C and 1035°C

Fig. S9a shows the average island area for both 1000°C and 1035°C with increasing growth time. The maxima for each temperature corresponds to the same time where we observe a graphene Raman spectra. Increasing the growth time beyond this shows a reduction in the average island area, which correlates to the Raman spectra no longer showing the presence of nanocrystalline graphite. The reduction in area could be due to one of many factors such as hydrogen etching of the graphene [2], the evaporation of the copper substrate under the graphene or a change in the concentration of surface carbon available for growth due to the exhaustion of the carbon [3].

Simultaneously we observe an increase in the average island separation for both temperatures, shown in Fig. S9b. The rate of separation after prolonged growth shows signs of slowing, but it is unclear if the separation will saturate like for the 950°C growths.

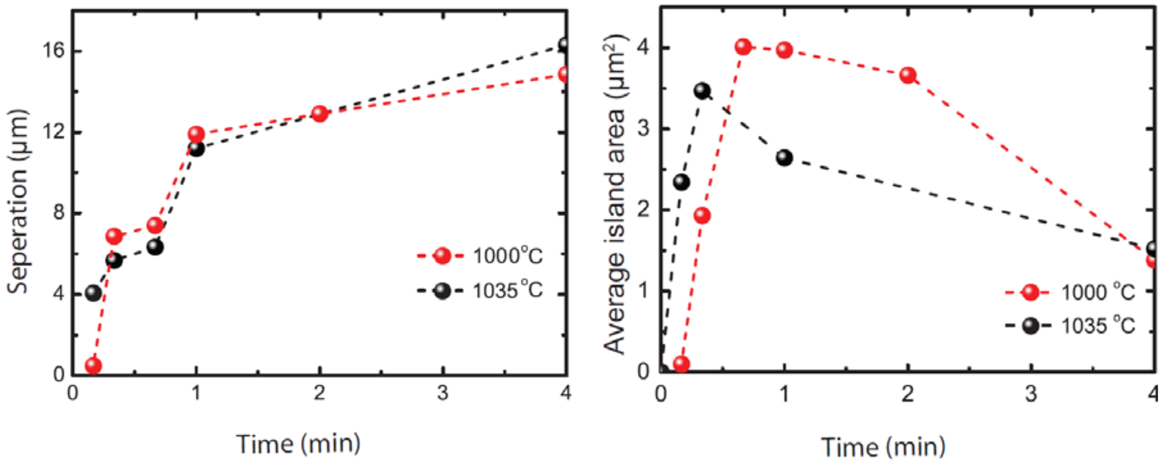

Figure S9 a) Shows the estimated island separation with increasing growth time for 1000°C and 1035°C, b) Shows the estimated average island size with increasing growth time for 1000°C and 1035°C.

Characterization of continuous graphene films

Fig. S10 shows an optical microscope image (Fig. S10a) and the Raman spectra (Fig. S10b) for three regions of continuous graphene grown using the two stage growth method and transferred on SiO_2/Si. The D-, G- and 2D- bands were fitted and used for the continuous growth data points which appear in Figure 1 in the main text. These continuous films were used to fabricate the Hall bar devices shown in Fig S10c (top). Fig. S10c shows the mapping of the Full width at half maximum (FWHM) of the 2D band (middle) and the intensity ratio of the D to G peak, I_D/I_G (bottom). The FWHM of the 2D band ranges from 30 to 35 cm^{-1} which is typical for CVD grown monolayer graphene. The Raman maps have been taken with 1μm step size.

On the continuous films we still observe a small D peak, which indicates defects. However, this peak is usually observed on CVD grown polycrystalline graphene films and it is believed that defects arise from the misalignment of the islands as they come together and coalesce into a continuous film. Indeed, when we grow graphene islands which are larger than the area probed by our Raman measurement (i.e. spot size of 5 μm diameter) we do not observe the presence of the D band as shown in figure S10d. Therefore the D band that appears in the Raman spectra of the films is due to the defects arising from the grain boundaries.

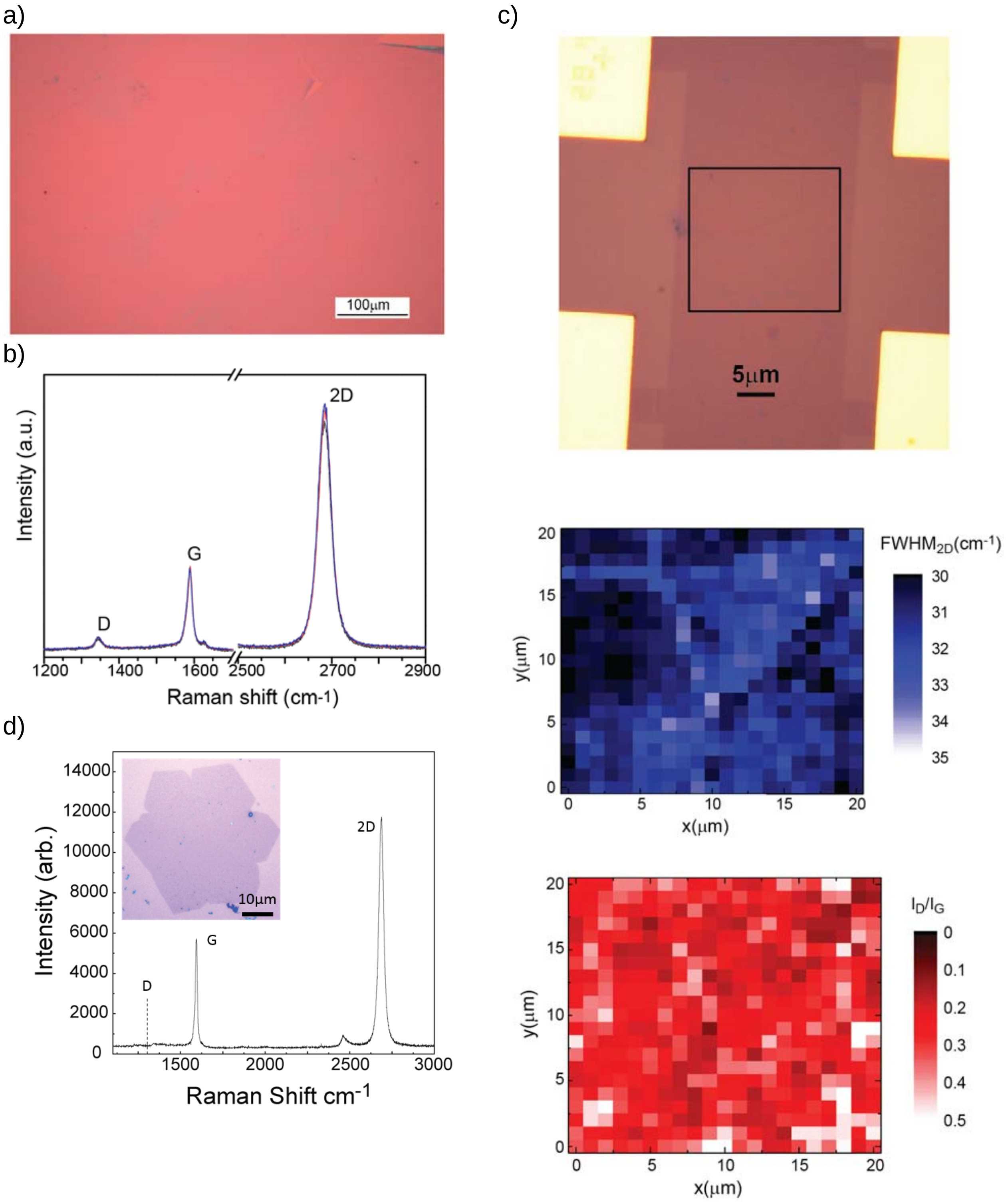

Figure S10. a) Optical image of the graphene film transferred on SiO_2/Si. The underlying substrate is visible in the upper right corner of the image. b) Three representative Raman spectra plotted for different regions of a continuous graphene grown using the two stage growth technique. c) Top: Optical image of a Hall bar device patterned from the graphene film shown in a). The yellow parts are the Au electrodes. The black square indicates the area used to map the Raman spectra shown in the middle and bottom. Middle: Full width at half maximum (FWHM) of the 2D band. Bottom: The intensity ratio of the D to G peak. d) Raman spectra of a large graphene island taken in the middle of the island. No defect-related D band is observed in this case.

Touch sensor fabrication and characterization

The touch sensor device was fabricated using a novel technique where all lithography is performed on the surface of a CVD graphene covered copper foil. Fig. S11 shows the outline of the fabrication process, while Fig. S12 shows images of key processing steps. CVD graphene on copper foils where coated in PMMA and contacts were defined using electron beam lithography, Fig. S11 a and b. The PMMA was developed shown in Fig. S12 a and metallized with 50 nm of gold, Fig. S11c and Fig. S12b. Strips of graphene were made between the contacts by coating the CVD graphene on copper foil with PMMA and defining a mask using electron beam lithography, Fig. S11d. The PMMA was developed and the exposed graphene was etched using Ar_2/O_2 reactive ion etching leaving conductive graphene channels between the gold contacts, Fig. S11e and Fig. S12d. The foil was then coated in PMMA again and wet etched in 1 molar $FeCl_3$ solution, Fig. S11f and Fig. S12e. The film was rinsed in ultra-pure water, Fig. S11g, and transferred to a clean PEN substrate, Fig. S11h and Fig. S12f. A PMMA dielectric layer was then coated on the graphene strips transferred to the PEN substrate. A second set of graphene strips were transferred on top of the PMMA/graphene/PEN. The top strips were rotated by 90 degrees with respect to the bottom graphene strips, giving the 2D network of capacitors for the touch sensor.

To characterize the contact and sheet resistance of the graphene films processed in this way we deposited gold contacts without etching graphene strips and transferred the films to a PEN substrate, set out in Fig. S111a-c, f-h.

The two terminal resistance of the graphene strips was measured in air using a probe station and a Keithley source-meter. The capacitance between graphene strips was measured using a Hameg 8118 LCR bridge with 1V AC excitation at 1KHz.

The two terminal resistance was measured as a function the number of squares (distance between probes divided by the sample width) shown in Fig. S12c. The fitted linear gradient is

representative of the film resistivity which we estimate to be 1.3KΩ/□, whereas the y intercept of the linear fit is the sum of the contact resistance for the two contacts, estimated to be 68 Ω for each contact.

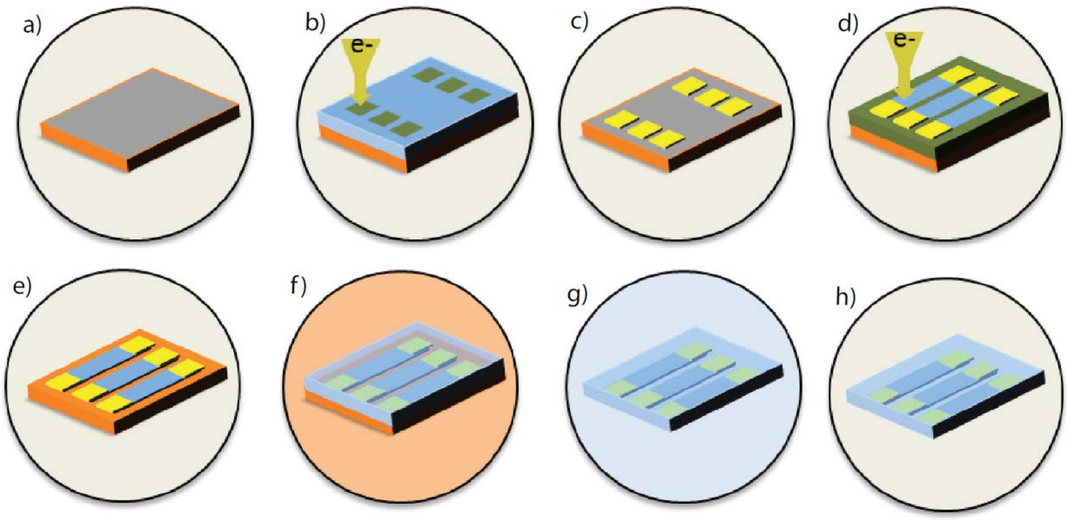

Figure S11. The process for fabricating the touch sensor devices. a) Graphene is grown on a copper substrate, b) The foil is coated with PMMA and contacts are exposed using electron beam lithography, c) Exposed regions are developed and metalized with 50nm of gold, d) The foil is coated with PMMA and an etch mask is defined between the gold contacts with electron beam lithography, e) Exposed graphene is etched using an Argon plasma, f)The foil is coated with PMMA and the copper is etched using 1 molar $FeCl_3$, g) The film is washed in ultra-pure water and h) the film is transferred to a PEN substrate.

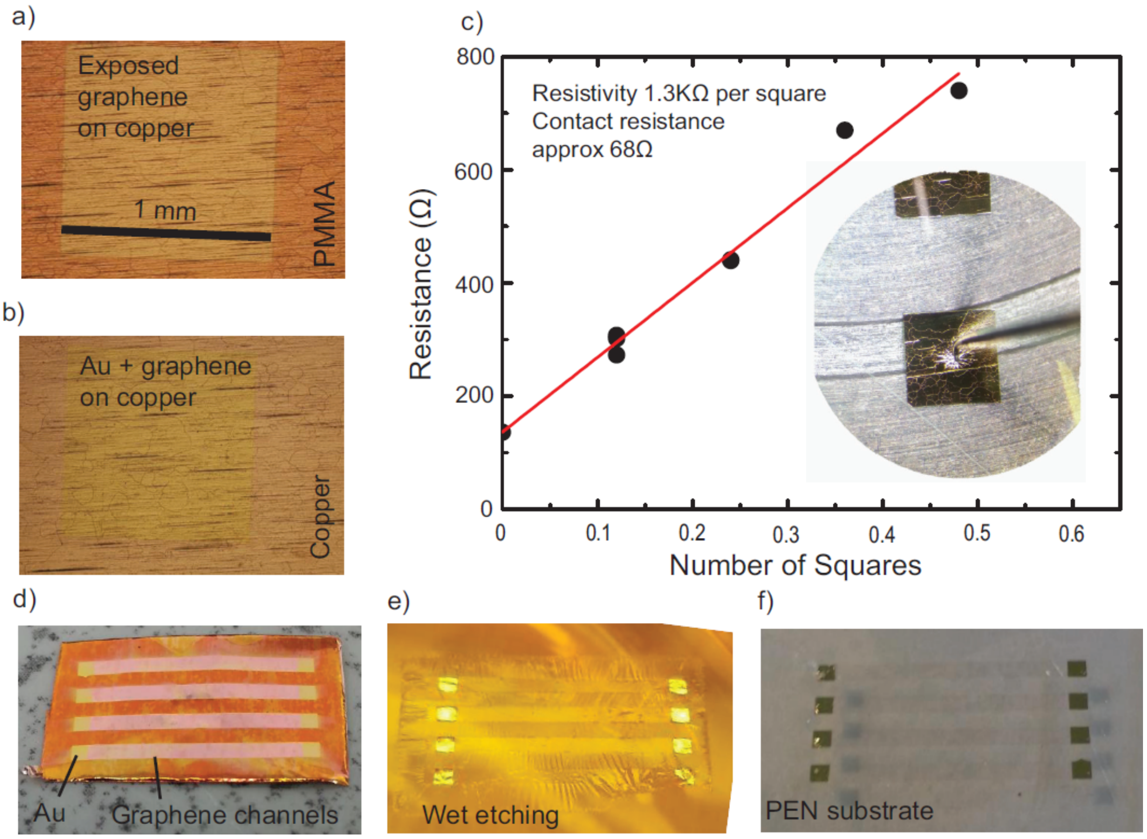

Figure S12. a) Shows a window in PMMA after electron beam exposure and development on copper foil coated in CVD graphene, b) Shows a gold square after the metallization gold on top of a copper foil coated with CVD graphene, c) shows the resistance for different separations of gold contacts on graphene transferred to a PEN substrate where the y intercept gives contact resistance and the fitted gradient gives the resistivity, d) Shows gold contacts connected by graphene strips on the surface of the copper foil, e) shows a gold contacts supported by a PMMA film in $FeCl_3$ etchant, f) shows the transferred structure onto a PEN substrate.

Costing of graphene growth

The estimation of the cost of graphene growth was performed making several assumptions. There are three main factors affecting the price of producing graphene, the cost of growth gases; the energy cost for achieving the temperatures for growth and the cost of the copper used for growths. These calculations do not consider the cost of growth equipment such as furnaces, flow controllers and quartz tube. The costs were only estimated for published papers that contain enough information to estimate growth cost and the quality area of the graphene.

Cost of Gases. The cost of growth gases was estimated by collating the total volume of each gas used from growth times and gas flow rates. The cost per unit volume was then estimated assuming the same price for a set volume of gas [4] allowing for the total cost to be estimated shown in Table S1.

Gas Type	Cost	Volume (m^3)	Cost per Volume £/(m^3)
Hydrogen N = 5.5	£276.12[4]	8.8	31.37
Methane N = 5.5	£889.97[4]	10	88.99
Argon N = 6	£339.43[4]	10.6	32.02

Table S1. The estimation of cost of each different growth gas in £/m^3

From each research article, gas flow rates and times were collated shown in Table S2. A typical growth consists of following stages: heating to growth temperature, anneal of the copper foils, graphene island nucleation and the growth stage. Summing the volume of gas used for each stage allowed for the estimation of the total volume of each gas used and the cost of each gas.

	Hydrogen		Methane		Argon		
Article	Volume (m^3)	Cost (£)	Volume (m^3)	Cost (£)	Volume (m^3)	Cost (£)	Total cost (£)
Li et. al.[6] (2011)	2.6×10^{-4}	0.0081	4.5×10^{-5}	0.0040	–	–	0.0121
Venugopal et. al.[14] (2011)	2.6×10^{-4}	0.0081	4.5×10^{-5}	0.0040	–	–	0.0121
Bae et. al.[7] (2010)	1.1×10^{-3}	0.0351	1.05×10^{-3}	0.0934	–	–	0.1285
Li et. al.[8] (2011)	3.2×10^{-4}	0.0102	2.8×10^{-3}	0.2491	–	–	0.2590
Liu et. al.[9] (2011)	4.3×10^{-4}	0.0130	1.6×10^{-5}	0.0150	1.54×10^{-2}	0.4940	0.5220
Sun et. al.[10] (2012)	3.3×10^{-4}	0.0104	1.5×10^{-4}	0.0048*	1.66×10^{-2}	0.5334	0.5486
Hao et. al[11] (2013)	9.8×10^{-3}	0.3059	8.3×10^{-4}	0.0738	–	–	0.3797
Chen et. al.[12] (2013)	3.9×10^{-3}	0.1220	5.85×10^{-5}	0.1186	–	–	0.2406
This work (2015)	7.5×10^{-6}	0.0002	3.59×10^{-5}	0.0031	–	–	0.0033

Table S2. The collation of the gas consumption for several graphene growth studies, for the estimation of the total cost of graphene growth gases. The cost for methane has been substituted for that of argon, as argon diluted methane was used**.**

Cost of Energy. To estimate the total energy consumption and cost of each growth process we collated for each different stage of the process, the total growth time, power draw and the cost of electricity in Table S3. The energy consumed during the growth process in a hot wall furnace was estimated assuming an MTI 1200X - 5L tube furnace [5]. The power consumption is assumed to be at maximum during the ramping to the growth temperature (6KW) and that the power consumption scales linearly as a function of temperature to a

maximum of 6KW at 1200°C. The energy consumed by plasma based cold walled furnace is estimated at 0.7 KW. The energy consumed by a resistively heated cold walled furnace [this work] is 0.3KW for the ramping to the growth temperature and assumed to scale linearly as a function of temperature to 0.3KW at 1200°C. The cost of electricity is estimated at £0.1352 per KWH [13].

	Heating up			Anneal and Growth			
Article	Time (hours)	Power (KW)	Cost (£)	Time (hours)	Power (KW)	Cost (£)	Total cost (£)
Li et. al.[6] (2009)	1.000	6.0	0.811	0.50	5.175	0.349	1.159
Venugopal et. al.[14] (2011)	1.000	6.0	0.811	0.50	5.175	0.349	1.159
Bae et. al.[7] (2010)	0.666	6.0	0.540	1.00	5.175	0.700	1.235
Li et. al.[8] (2011)	0.666	6.0	0.540	1.50	5.175	1.049	1.590
Liu et. al.[9] (2011)	0.167	6.0	0.135	0.55	5.175	0.384	0.520
Sun et. al.[10] (2012)	-	-	-	0.22	0.700	0.021	0.021
Hao et. al[11] (2013)	0.666	6.0	0.540	15.08	5.175	10.551	11.091
Chen et. al.[12] (2013)	0.666	6.0	0.540	6.30	5.175	4.408	4.948
This work (2015)	0.050	0.3	0.002	0.26	0.195	0.007	0.009

Table S3. The collation energy consumption of each growth procedure, broken down into the heating of the foils to the growth temperature and the growth process.

Cost of Copper. We assumed 1cm^2 of 25μm thick copper was used in a growth. The cost of copper (99.999 %) is £88.20 for 250cm^2 giving a cost for 1cm^2 of £0.3528.

Estimation of total price. By summing the cost of the growth gases, energy used and the cost of the copper foils we can estimate the total cost of graphene production, shown in Table S4. It is clear from Table S4 that the lowest cost of production is this work and that the limiting factor is the cost of the copper foils used in the growth.

	Cost (£)			
Article	Gas cost	Energy cost	Copper cost	Total cost
Li et. al.[6] (2009); Venugopal et. al.[14] (2011)	0.2590	1.159	0.3528	1.762
Bae et. al.[7] (2009)	0.1285	1.235	0.3528	1.716
Li et. al.[8] (2011)	0.0121	1.590	0.3528	1.955
Liu et. al.[9] (2011)	0.5220	0.520	0.3528	1.35
Sun et. al.[10] (2012)	0.5486	0.021	0.3528	0.922
Hao et. al[11] (2013)	0.3797	11.091	0.3528	11.820
Chen et. al.[12] (2013)	0.2406	4.948	0.3528	5.428
This work (2015)	0.0033	0.009	0.3528	0.365

Table S4. The estimation of cost of each price component and the total cost of the growth for each article in £.

Electronic Quality Factor estimation

To determine the electronic quality factor for each article, the reported field effect mobility was used and the area of the device was estimated from dimensions given or images appearing in the articles. The data was collated into Table S5 and are shown in Figure S11.

Article	Mobility ($cm^2/(Vs)$)	Area (μm^2)	Electronic Quality Factor (Q)
Li et. al.[6] (2009)	4050	69.66	2.82×10^5
Bae et. al.[7] (2009)	3175	1.16	3.68×10^3
Li et. al.[8] (2011)	4000	–	–
Liu et. al.[9] (2011)	3600	0.59	2.13×10^3
Venugopal et. al.[14] (2011)	2450	2880	7×10^6
Sun et. al.[10] (2012)	1800	8	1.44×10^4
Hao et. al[11] (2013)	10800	3.9	4.21×10^4
Chen et. al.[12] (2013)	5200	64.9	3.33×10^5
This work (2015)	2351	2500	5.87×10^6
This work (2015)	1936	3750	7.2×10^6
This work (2015)	1744	2500	4.36×10^6
This work (2015)	3300	1250	4.13×10^6

Table S5. The collation of information required to make the estimation of Electronic Quality Factor for each article. The mobility is the field effect mobility (cm^2/Vs), the area is the device area (μm^2) over which the mobility was estimated and the Electronic Quality Factor ($\mu m^2 x cm^2/(Vs)$).

Plotting the price *versus* the mobility shown in Fig. S11a demonstrates the general trend of the cost of production with respect to the mobility. The cost of producing graphene using a cold wall furnace reduces the price significantly when compared to graphene produced in a hot wall furnace while not impacting on the quality of the graphene produced as the general trend would imply.

The quality of cold-wall CVD graphene as compared to that grown with other methods is better assessed using the electronic quality factor (Q) that. As shown in the Figure S11b, graphene grown by resistive heating cold-wall CVD has Q ranging from $4x10^6$ to $7.2x10^6$, whereas most reports of monolayer graphene grown by hot-wall CVD have Q ranging from 10^3 to 7×10^6. This demonstrates the enhanced electronic quality range of graphene grown by

resistive heating cold-wall CVD over the reported values of monolayer graphene grown by hot-wall CVD. Thus as shown in Figure S11b the cold-wall CVD provides a method to produce high quality graphene at a much lower cost than hot-wall CVD. Employing this method in industry will reduce also the retail price of graphene which currently is as high as 21£/cm^2 as shown in Figure S11c.

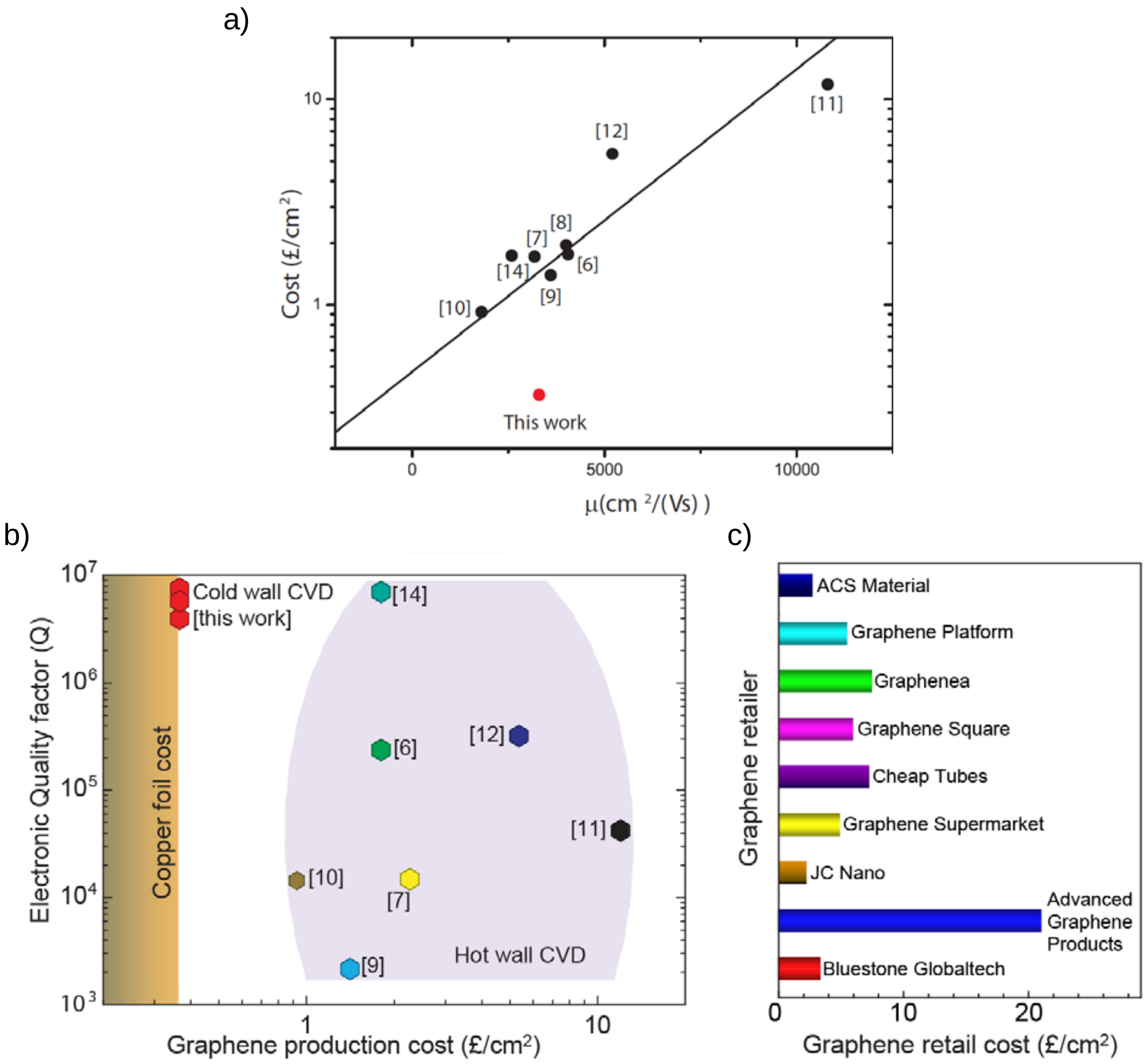

Figure S11. a) A plot of the price of graphene production per cm^2 against the measured mobility. The general trend is a linear fit of the data omitting the data point from this work. b) Estimated cost for different CVD growth processes for monolayer-graphene on Cu plotted against the electronic quality factor, Q. c) Retail cost of monolayer graphene as of April 2015 taken from the website of different suppliers of monolayer graphene grown by CVD on Cu.

References for the Supporting Information

[1] Matlab - Image processing toolbox -http://www.mathworks.co.uk/help/images/image-enhancement-and-analysis.html

[2] Y. Zhang, Z. Li, P. Kim, L. Zhang, C. Zhou, *ACS Nano* **6**, 126-132 (**2011)**.

[3] H. Kim, C. Mattevi, M. R. Calvo, J. C. Oberg, L. Artiglia, C. F. Hirjibehedin, M. Chhowalla, E. Saiz, *ACS Nano* **6**, 3614-3623 (2012).

[4] Costs and Volumes taken from BOC LTD website- http://www.Boconline.co.uk - Dec 2013

[5] Specifications taken from MTI website for MTI 1200X - 5L split tube furnace - http://www.mtixtl.com - Dec 2013

[6] X. Li, W. Cai, J. An, S. Kim, J. Nah, D. Yang, R. Piner, A. Velamakanni, I. Jung, E. Tutuc, S. K. Banerjee, L. Colombo, R. S. Ruoff, *Science* **324**, 1312 (2009).

[7] S. Bae, H Kim, Y. Lee, X. Xu, J. S. Park, Y. Zheng, J. Balakrishnan, T. Lei, H. R. Kim, Y. I. Song, Y. J. Kim, K. S. Kim, B. Ozyilmaz, J. H. Ahn, B. H. Hong, S. Iijima, *Nature Nanotech* **5**, 574 (2010).

[8] Li, X.; Magnuson, C. W.; Venugopal, A.; Tromp, R. M.; Hannon, J. B.; Vogel, E. M.; Colombo, L.; Ruoff, R. S.; *J. Am. Chem. Soc.*, **133**, 2816-2819 (2011**)**.

[9] W. Liu, H. Li, C. Xu, Y. Khatami, K Banerjee, *Carbon* **49** , 4122 (2011).

[10] J. Sun, N. Lindvall, M. T. Cole, K. T. T. Angel, W. Teng, K. B. K. Teo, D. H. C. Chua, J. Liu, A. Yurgens, *TNANO* **11**, 255 (2012).

[11] Y. Hao, M. S. Bharathi, L. Wang, Y. Liu, H. Chen, S. Nie, X. Wang, H. Chou, C. Tan, B. Fallahazad, H. Ramanarayan, C. W. Magnuson, E. Tutuc, B. I. Yakobson, K. F. McCarty, Y. W. Zhang, P. Kim, J. Hone, L. Colombo, R. S. Ruoff, *Science* **342**, 720 (2013).

[12] S. Chen, H. Ji, H. Chou, Q. Li, H. Li, J. W. Suk, R. Piner, L. Liao, W. Cai, R. S. Ruoff, *Adv. Mater.* **25**, 2062 **(**2013).

[13] Based on December 2013 Energy saving trust estimation - http://www.energysavingtrust.org.uk/Energy-Saving-Trust/Our-calculations

[14] Venugopal, A. et al. Effective mobility of single-layer graphene transistors as a function of channel dimensions, *J. Appl. Phys.* **109**, 104511 (2011).
